# Supplementary material for: scMD facilitates cell type deconvolution using single-cell DNA methylation references
Source: Commun Biol. 2024 Jan 2;7:1. doi: 10.1038/s42003-023-05690-5 (PMC10762261; doi:10.1038/s42003-023-05690-5)
Supplement: Supplementary file 2 — Supplementary Information [file 42003_2023_5690_MOESM2_ESM.pdf]

Supplementary Information

Supplementary Algorithm

Supplementary Algorithm 1: The scMD algorithm

Notation:

I - Number of bulk CpG sites; $\mathcal{I}$ - Set of bulk CpG sites (450k/850k/25M)
S - Number of bulk samples
P - Number of scDNAm DNAm sites; $\mathcal{P}$ - Set of scDNAm DNAm sites
C - Number of scDNAm cells

Input: Bulk DNAm data (beta values) $\boldsymbol{Y}_{I\times S}$, scDNAm methylated counts $\boldsymbol{M}_{P\times C}$, unmethylated counts $\boldsymbol{U}_{P\times C}$.

Output: scMD estimated cellular fractions $\hat{\boldsymbol{W}}$.

1. Reduce feature-wise dimension:
   - Determine the intersection set of CpGs: $\mathcal{G} = \mathcal{I} \cap \mathcal{P}$, resulting in G total CpG sites.
   - Update matrices: $\boldsymbol{Y}_{G\times S} \leftarrow \boldsymbol{Y}_{I\times S}$, $\boldsymbol{M}_{G\times C} \leftarrow \boldsymbol{M}_{P\times C}$, $\boldsymbol{U}_{G\times C} \leftarrow \boldsymbol{U}_{P\times C}$.
2. Reduce cell-wise dimension: For all $k \in K$ cell types, aggregate cells within type k for $\boldsymbol{M}_{G\times C}$ and $\boldsymbol{U}_{G\times C}$. Update: $\boldsymbol{M}_{G\times K} \leftarrow \boldsymbol{M}_{G\times C}$, $\boldsymbol{U}_{G\times K} \leftarrow \boldsymbol{U}_{G\times C}$.
3. Identify marker CpGs: Conduct two-sided Fisher's exact tests to distinguish cell type k from the other cell types for each DNAm site $g \in \mathcal{G}$ with $\boldsymbol{M}_{G\times K}$ and $\boldsymbol{U}_{G\times K}$.
4. Construct scDNAm signature: Choose the top n marker CpG sites per cell type as final markers. Update: $\boldsymbol{Y}_{nK\times S} \leftarrow \boldsymbol{Y}_{G\times S}$, $\boldsymbol{M}_{nK\times K} \leftarrow \boldsymbol{M}_{G\times K}$, $\boldsymbol{U}_{nK\times K} \leftarrow \boldsymbol{U}_{G\times K}$. Calculate signature beta matrix: $\boldsymbol{B}_{nK\times K} = \boldsymbol{M}_{nK\times K}/(\boldsymbol{U}_{nK\times K} + \boldsymbol{M}_{nK\times K})$.
5. Estimate cellular fractions: Compute $\hat{\boldsymbol{W}}$ with $\boldsymbol{Y}_{nK\times S}$ and $\boldsymbol{B}_{nK\times K}$ using EnsDeconv.

Supplementary Figures

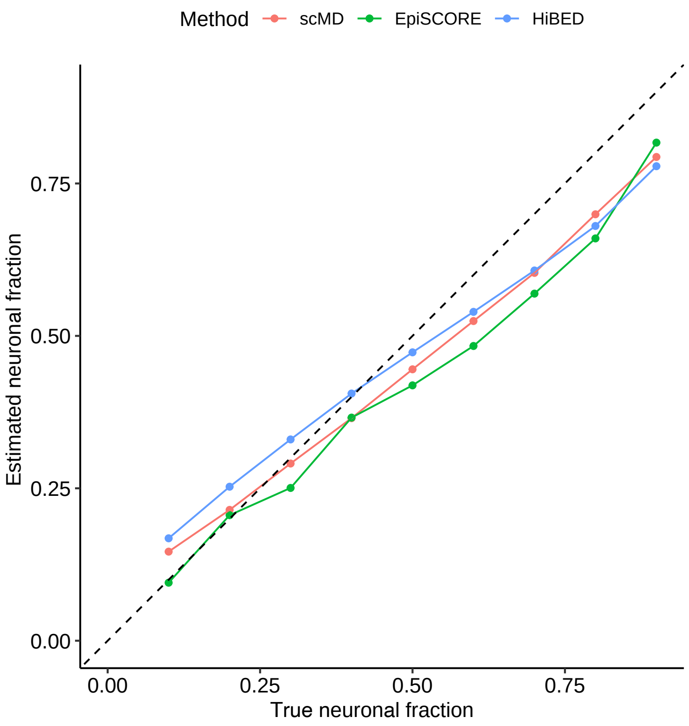

Supplementary Figure 1: Comparative analysis of neuron proportions estimated by scMD, EpiSCORE, and HiBED versus mixture neuron proportions with the Guintivano data[1]. MAE is 0.060, 0.072, and 0.064, respectively, for scMD, EpiSCORE, and HiBED.

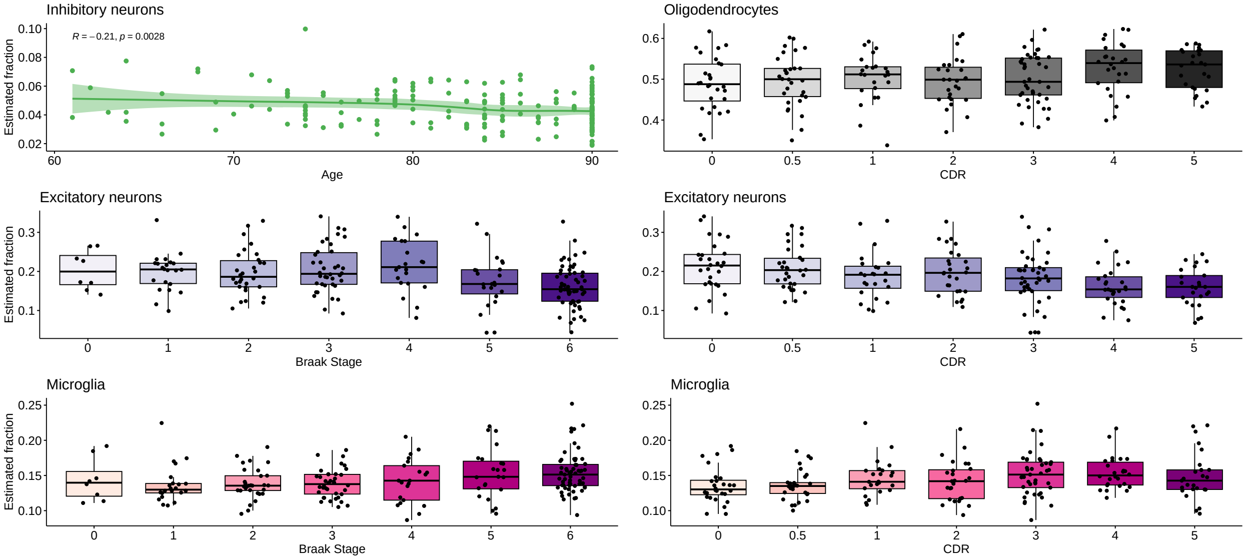

Supplementary Figure 2: Identifying differential cellular fractions with the Mount Sinai Brain Bank (MSBB) data[2] using HiBED. For inhibitory neurons, the scatterplot is presented with a shaded area representing the confidence interval around the LOESS smooth line. For all box plots, the median is indicated by the central line, quartiles by the box edges, and whiskers extend to 1.5 times the interquartile range, with outliers plotted individually.

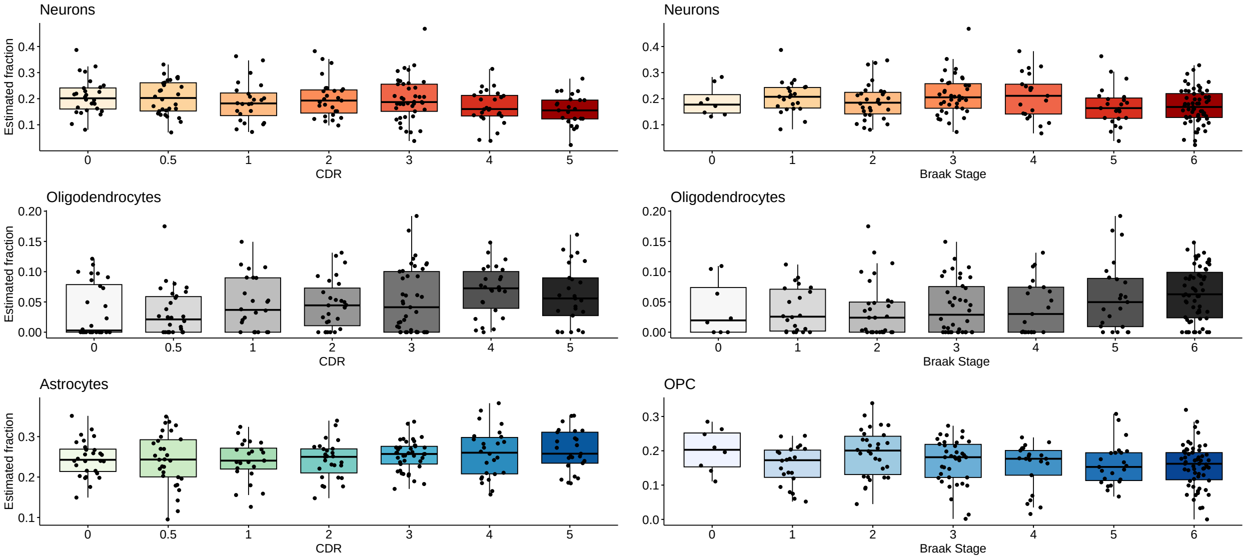

Supplementary Figure 3: Identifying differential cellular fractions with the MSBB[2] data using EpiSCORE. For all box plots, the median is indicated by the central line, quartiles by the box edges, and whiskers extend to 1.5 times the interquartile range, with outliers plotted individually.

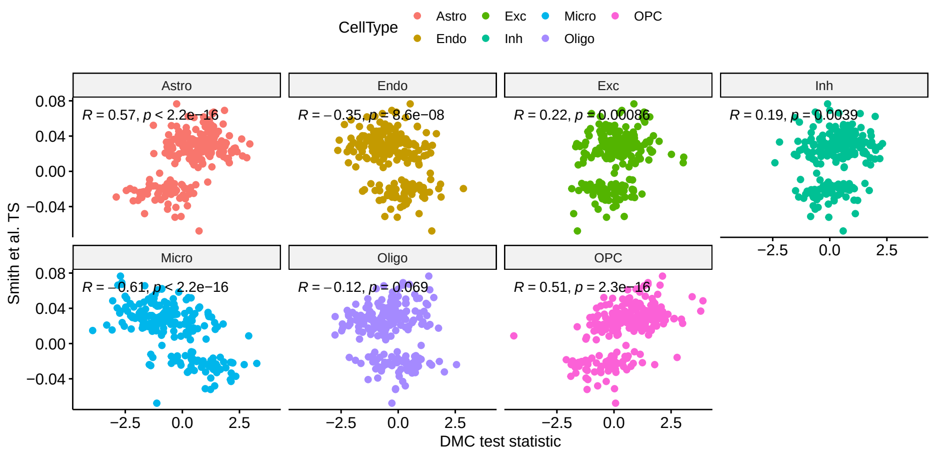

Supplementary Figure 4: Scatterplots depicting the relationship between the DMC test statistics from our CTS analysis and those reported in the bulk analysis by [3].

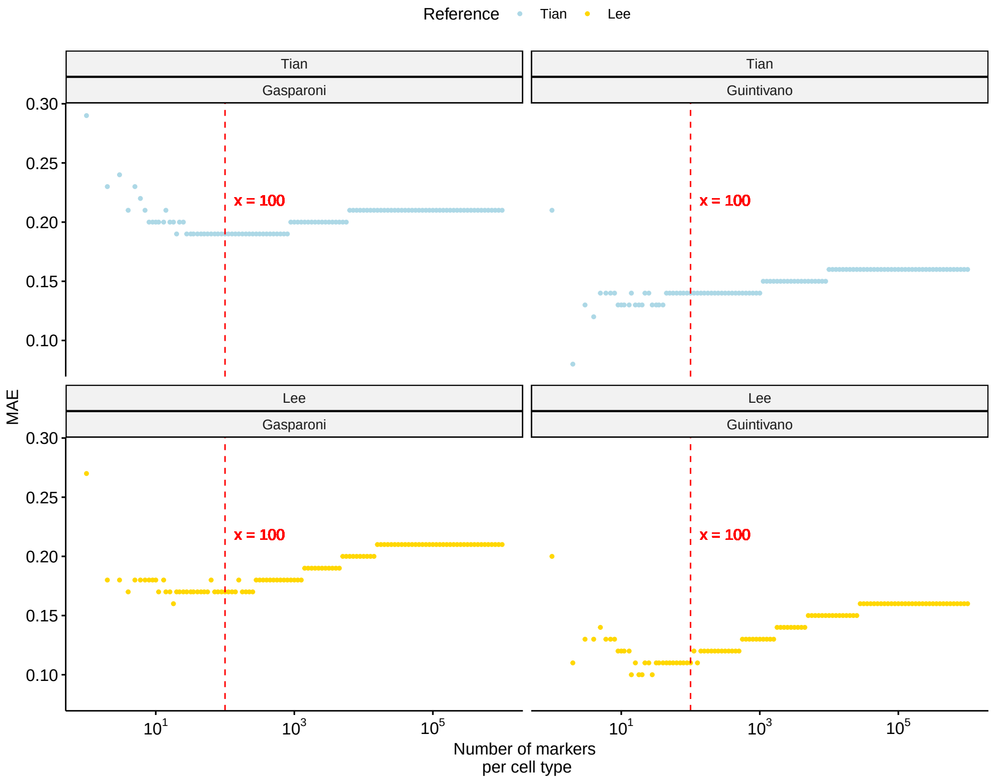

Supplementary Figure 5: The mean absolute error (MAE) between estimated DNAm fraction and measured DNAm fraction as a function of different numbers of markers per cell type. Each row panel is a reference dataset, and each column panel is a bulk dataset.

Supplementary Tables

Supplementary Table 1: Number of DNAm sites of scDNAm datasets during the process before generating the signatures. The cluster-level CpGs are then matched to bulk data and result in 450k, 850k, or 25 million (for WGBS) CpGs. We merged +/- strands when considering CG alone.

	single-cell CG+CH	single-cell CG	cluster-level CG
Lee[4] (mean (sd))	48,216,050 (49,294,600)	2,244,381 (808,865)	27,147,236 (177,359)
Tian[5] (mean (sd))	NA	NA	27,107,054 (579,356)

Supplementary Table 2: Typical GREAT output for cell type-specific CpG markers.

Cell type	Significant process
Excitatory neuron	positive regulation of excitatory postsynaptic potential
	modulation of excitatory postsynaptic potential
Inhibitory neuron	cerebral cortex GABAergic interneuron fate commitment
	cerebral cortex GABAergic interneuron differentiation
	GABAergic neuron differentiation
	negative regulation of synaptic transmission, GABAergic
	interneuron axon guidance
Microglia	macrophage chemotaxis
	macrophage migration
Oligodendrocyte	oligodendrocyte differentiation
	oligodendrocyte development

Supplementary Table 3: Summary of sorted-cell and bulk datasets for validation. The first three datasets are based on sorted cells, while the rest are bulk datasets.

Dataset	Technical platform	Cell types (Sample size)
Mendizabal[6]	WGBS	neurons (25) and oligodendrocyte (20)
Guintivano[1]	Illumina 450k	neurons (29) and non-neurons (29)
		Bulk (9)
Gasparoni[7]	Illumina 450k	neurons (31) and non-neurons (31)
ROS (Religious Orders Study)[8]	Illumina 450k	Bulk (49)
NAc (Nucleus Accumbens)[9]	Illumina EPIC & RNA-seq	Bulk (211)
MSBB (Mount Sinai Brain Bank)[2]	Illumina EPIC	Bulk (201)

Supplementary Table 4: MAE for different benchmarking datasets and deconvolution method.

MAE	scMD	EpiSCORE	HiBED
Guitinvano purified samples	0.09	0.11	0.10
Guitinvano mixed samples	0.060	0.072	0.064
Gasparoni	0.11	0.13	0.14
ROS	0.07	0.14	0.13

Supplementary Table 5: Friedman-Nemenyi posthoc test results. It is underpowered, given a small number of observations.

p.value	scMD	EpiSCORE
EpiSCORE	0.036	-
HiBED	0.181	0.759

Supplementary Table 6: Comparison of absolute errors in estimated vs. measured cell type fractions for ROS data. One-sided Diebold-Mariano test results for scMD, HiBED, and EpiSCORE. The alternative hypotheses tested are: scMD outperforms HiBED in accuracy; scMD outperforms EpiSCORE in accuracy; and HiBED outperforms EpiSCORE in accuracy.

p.value	scMD	EpiSCORE
EpiSCORE	$< 2.2e-16$	-
HiBED	$< 2.2e-16$	4.624e-05

Supplementary Table 7: Cell-type-specific (CTS) differential fraction analyses of different phenotypes in MSBB bulk data[2] using scMD, EpiSCORE, and scMD estimated fractions. Correlation coefficients and p-values for phenotype-fraction associations in different cell types are provided.

		Correlation (p-value)							
Phenotype	Method	Astro	Micro	Endo	Oligo	OPC	Inh	Exc	Neuron
Age	scMD	0.078 (0.269)	0.113 (0.110)	-0.034 (0.634)	0.061 (0.389)	-0.174 (0.013)	-0.107 (0.129)	-0.045 (0.523)	-0.071 (0.318)
	EpiSCORE	-0.030 (0.675)	0.048 (0.501)	0.065 (0.357)	0.104 (0.140)	-0.059 (0.409)			-0.047 (0.506)
	HiBED	-0.046 (0.514)	0.096 (0.177)	0.048 (0.495)	0.049 (0.493)		-0.201 (0.043)	-0.058 (0.414)	-0.092 (0.192)
CDR	scMD	0.035 (0.621)	0.205 (0.003)	-0.089 (0.207)	0.215 (0.002)	-0.204 (0.004)	-0.138 (0.051)	-0.285 (0.000)	-0.245 (0.000)
	EpiSCORE	0.150 (0.034)	0.138 (0.051)	0.039 (0.579)	0.236 (0.001)	-0.095 (0.180)			-0.207 (0.003)
	HiBED	-0.099 (0.161)	0.167 (0.018)	-0.065 (0.360)	0.197 (0.005)		0.046 (0.513)	-0.294 (0.000)	-0.267 (0.000)
CERAD	scMD	0.003 (0.963)	0.024 (0.740)	-0.016 (0.825)	-0.038 (0.592)	0.052 (0.467)	0.024 (0.738)	0.026 (0.713)	0.027 (0.708)
	EpiSCORE	0.106 (0.244)	-0.018 (0.799)	0.015 (0.833)	0.052 (0.466)	-0.114 (0.107)			0.070 (0.325)
	HiBED	-0.012 (0.870)	0.059 (0.404)	0.018 (0.800)	-0.061 (0.386)		0.0477 (0.508)	0.038 (0.594)	0.035 (0.619)
Braak	scMD	0.039 (0.583)	0.214 (0.002)	-0.037 (0.600)	0.117 (0.097)	-0.039 (0.581)	-0.096 (0.175)	-0.241 (0.001)	-0.200 (0.005)
	EpiSCORE	0.063 (0.374)	0.070 (0.327)	0.134 (0.059)	0.233 (0.001)	-0.145 (0.040)			-0.172 (0.015)
	HiBED	-0.020 (0.778)	0.202 (0.004)	-0.011 (0.876)	0.108 (0.126)		0.100 (0.015)	-0.232 (0.015)	-0.211 (0.003)

Supplementary Table 8: Number of identified differentially methylated cytosines (DMCs) in individual cell types using CellDMC and scMD estimated cellular fractions, with FDR < 0.05. Covariates sex and race are adjusted in all analyses.

	Number of DMCs						
	Astro	Micro	Endo	Oligo	OPC	Inh	Exc
Age	12	22			57	2	1
CDR	195		2				
CERAD				1			
Braak		4	3			55	1

Supplementary Table 9: DMCs close to GWAS loci by phenotype and cell type. Following the definition of GWAS loci[10], for our analysis, differential sites situated within a distance of less than 500 kb from a GWAS SNP were considered to be close. For a more granular breakdown of these sites, please refer to Supplementary Data 3.

	Astro	Micro	Endo	Oligo	OPC	Inh	Exc
Age	9	11		5	31	11	
CDR	102						
Braak		2	2			30	1

Supplementary References

1. Guintivano, J., Aryee, M. J. & Kaminsky, Z. A. A cell epigenotype specific model for the correction of brain cellular heterogeneity bias and its application to age, brain region and major depression. *Epigenetics* **8,** 290–302 (2013).
2. Wang, M., Beckmann, N. D., Roussos, P., Wang, E., Zhou, X., Wang, Q., Ming, C., Neff, R., Ma, W., Fullard, J. F., *et al.* The Mount Sinai cohort of large-scale genomic, transcriptomic and proteomic data in Alzheimer's disease. *Scientific data* **5,** 1–16 (2018).
3. Smith, R. G., Pishva, E., Shireby, G., Smith, A. R., Roubroeks, J. A., Hannon, E., Wheildon, G., Mastroeni, D., Gasparoni, G., Riemenschneider, M., *et al.* A meta-analysis of epigenome-wide association studies in Alzheimer's disease highlights novel differentially methylated loci across cortex. *Nature communications* **12,** 3517 (2021).
4. Lee, D.-S., Luo, C., Zhou, J., Chandran, S., Rivkin, A., Bartlett, A., Nery, J. R., Fitzpatrick, C., O'Connor, C., Dixon, J. R., *et al.* Simultaneous profiling of 3D genome structure and DNA methylation in single human cells. *Nature methods* **16,** 999–1006 (2019).
5. Tian, W., Zhou, J., Bartlett, A., Zeng, Q., Liu, H., Castanon, R. G., Kenworthy, M., Altshul, J., Valadon, C., Aldridge, A., *et al.* Single-cell DNA methylation and 3D genome architecture in the human brain. *Science* **382,** eadf5357 (2023).
6. Mendizabal, I., Berto, S., Usui, N., Toriumi, K., Chatterjee, P., Douglas, C., Huh, I., Jeong, H., Layman, T., Tamminga, C. A., *et al.* Cell type-specific epigenetic links to schizophrenia risk in the brain. *Genome biology* **20,** 1–21 (2019).
7. Gasparoni, G., Bultmann, S., Lutsik, P., Kraus, T. F., Sordon, S., Vlcek, J., Dietinger, V., Steinmaurer, M., Haider, M., Mulholland, C. B., *et al.* DNA methylation analysis on purified neurons and glia dissects age and Alzheimer's disease-specific changes in the human cortex. *Epigenetics & chromatin* **11,** 1–19 (2018).
8. De Jager, P. L., Srivastava, G., Lunnon, K., Burgess, J., Schalkwyk, L. C., Yu, L., Eaton, M. L., Keenan, B. T., Ernst, J., McCabe, C., *et al.* Alzheimer's disease: early alterations in brain DNA methylation at ANK1, BIN1, RHBDF2 and other loci. *Nature neuroscience* **17,** 1156–1163 (2014).

9. Markunas, C. A., Semick, S. A., Quach, B. C., Tao, R., Deep-Soboslay, A., Carnes, M. U., Bierut, L. J., Hyde, T. M., Kleinman, J. E., Johnson, E. O., *et al.* Genome-wide DNA methylation differences in nucleus accumbens of smokers vs. nonsmokers. *Neuropsychopharmacology* **46,** 554–560 (2021).

10. Bellenguez, C., Küçükali, F., Jansen, I. E., Kleineidam, L., Moreno-Grau, S., Amin, N., Naj, A. C., Campos-Martin, R., Grenier-Boley, B., Andrade, V., *et al.* New insights into the genetic etiology of Alzheimer's disease and related dementias. *Nature genetics* **54,** 412–436 (2022).
