# Supplementary material for: scMD facilitates cell type deconvolution using single-cell DNA methylation references
Source: Commun Biol. 2024 Jan 2;7:1. doi: 10.1038/s42003-023-05690-5 (PMC10762261; doi:10.1038/s42003-023-05690-5)
Supplement: Supplementary file 3 — Description of Additional Supplementary Files [file 42003_2023_5690_MOESM3_ESM.pdf]

Description of Additional Supplementary Files

File name: Supplementary Data 1
Description: GREAT analysis result for cell type-specific markers in scMD

File name: Supplementary Data 2
Description: CellDMC result using scMD estimated fractions

File name: Supplementary Data 3
Description: Cell-type specific (CTS) DMCs close to GWAS loci by phenotype and cell type within 500 kb

File name: Supplementary Data 4
Description: Enrichment analysis result for CellDMC result with gprofiler2

File name: Supplementary Data 5
Description: Source data for Figure 2a

File name: Supplementary Data 6
Description: Source data for Figure 2b

File name: Supplementary Data 7
Description: Source data for Figure 2c

File name: Supplementary Data 8
Description: Source data for Figure 3

File name: Supplementary Data 9
Description: Source data for Figure 5b-h
