# Supplementary material for: scMD facilitates cell type deconvolution using single-cell DNA methylation references
Source: Commun Biol. 2024 Jan 2;7:1. doi: 10.1038/s42003-023-05690-5 (PMC10762261; doi:10.1038/s42003-023-05690-5)
Supplement: Supplementary file 5 — Reporting Summary [file 42003_2023_5690_MOESM5_ESM.pdf]

nature portfolio

Corresponding author(s): Jiebiao Wang

Last updated by author(s): Oct 18, 2023

Reporting Summary

Nature Portfolio wishes to improve the reproducibility of the work that we publish. This form provides structure for consistency and transparency in reporting. For further information on Nature Portfolio policies, see our Editorial Policies and the Editorial Policy Checklist.

Statistics

For all statistical analyses, confirm that the following items are present in the figure legend, table legend, main text, or Methods section.

n/a	Confirmed	
☐	☒	The exact sample size (*n*) for each experimental group/condition, given as a discrete number and unit of measurement
☐	☒	A statement on whether measurements were taken from distinct samples or whether the same sample was measured repeatedly
☐	☒	The statistical test(s) used AND whether they are one- or two-sided<br>*Only common tests should be described solely by name; describe more complex techniques in the Methods section.*
☐	☒	A description of all covariates tested
☐	☒	A description of any assumptions or corrections, such as tests of normality and adjustment for multiple comparisons
☐	☒	A full description of the statistical parameters including central tendency (e.g. means) or other basic estimates (e.g. regression coefficient) AND variation (e.g. standard deviation) or associated estimates of uncertainty (e.g. confidence intervals)
☒	☐	For null hypothesis testing, the test statistic (e.g. *F*, *t*, *r*) with confidence intervals, effect sizes, degrees of freedom and *P* value noted<br>*Give P values as exact values whenever suitable.*
☒	☐	For Bayesian analysis, information on the choice of priors and Markov chain Monte Carlo settings
☒	☐	For hierarchical and complex designs, identification of the appropriate level for tests and full reporting of outcomes
☐	☒	Estimates of effect sizes (e.g. Cohen's *d*, Pearson's *r*), indicating how they were calculated

Our web collection on statistics for biologists contains articles on many of the points above.

Software and code

Policy information about availability of computer code

Data collection	No software was used for data collection since we used public available datasets.
Data analysis	We utilized the following software, all of which are available on GitHub or Bioconductor:<br>EnsDeconv (v0.2.0), minfi (v1.46.0), EpiDISH (v2.14.1), HiBED (v0.99.15), gprofiler2 (v0.2.2), and scMD (v0.2.0).<br>GREAT is from https://great.stanford.edu/.

For manuscripts utilizing custom algorithms or software that are central to the research but not yet described in published literature, software must be made available to editors and reviewers. We strongly encourage code deposition in a community repository (e.g. GitHub). See the Nature Portfolio guidelines for submitting code & software for further information.

Data

Policy information about availability of data

All manuscripts must include a data availability statement. This statement should provide the following information, where applicable:

- Accession codes, unique identifiers, or web links for publicly available datasets
- A description of any restrictions on data availability
- For clinical datasets or third party data, please ensure that the statement adheres to our policy

• The Lee scDNAm data can be obtained through (https://www.ncbi.nlm.nih.gov/geo/query/acc.cgi?acc=GSE130711).

• The Tian scDNAm data is available at http://neomorph.salk.edu/wtian/hba-data/.
• The Guintivano DNAm data can be downloaded from a Bioconductor package named FlowSorted.DLPFC.450k.
• The Gasparoni DNAm data is publicly available on (https://www.ncbi.nlm.nih.gov/geo/query/acc.cgi?acc=GSE66351).
• The Mendizabal DNAm data is publicly available on (https://www.ncbi.nlm.nih.gov/geo/query/acc.cgi?acc=GSE108066).
• The NAc bulk DNAm and RNA data are publicly available on (https://www.ncbi.nlm.nih.gov/geo/query/acc.cgi?acc=GSE147040) and (https://www.ncbi.nlm.nih.gov/geo/query/acc.cgi?acc=GSE171936).
• The ROS and MSBB bulk data and related clinical data are publicly available on AD Knowledge Portal (https://adknowledgeportal.synapse.org/).

Research involving human participants, their data, or biological material

Policy information about studies with human participants or human data. See also policy information about sex, gender (identity/presentation), and sexual orientation and race, ethnicity and racism.

Reporting on sex and gender	NA
Reporting on race, ethnicity, or other socially relevant groupings	NA
Population characteristics	NA
Recruitment	NA
Ethics oversight	NA

Note that full information on the approval of the study protocol must also be provided in the manuscript.

Field-specific reporting

Please select the one below that is the best fit for your research. If you are not sure, read the appropriate sections before making your selection.

☒ Life sciences ☐ Behavioural & social sciences ☐ Ecological, evolutionary & environmental sciences

For a reference copy of the document with all sections, see nature.com/documents/nr-reporting-summary-flat.pdf

Life sciences study design

All studies must disclose on these points even when the disclosure is negative.

Sample size	Sample size was determined by previous published datasets which were used for our study.
Data exclusions	No experimental data was generated in our study and no data were excluded from analysis.
Replication	No replication of experimental data was performed since we did not collect any experimental data.
Randomization	Randomization of samples is not applicable in our study since we did not collect any experimental data.
Blinding	Blinding is not relevant to our study since we report scDNAm signature generation as main finding.

Reporting for specific materials, systems and methods

We require information from authors about some types of materials, experimental systems and methods used in many studies. Here, indicate whether each material, system or method listed is relevant to your study. If you are not sure if a list item applies to your research, read the appropriate section before selecting a response.

Materials & experimental systems

n/a	Involved in the study	
☒	☐	Antibodies
☒	☐	Eukaryotic cell lines
☒	☐	Palaeontology and archaeology
☒	☐	Animals and other organisms
☒	☐	Clinical data
☒	☐	Dual use research of concern
☒	☐	Plants

Methods

n/a	Involved in the study	
☒	☐	ChIP-seq
☒	☐	Flow cytometry
☒	☐	MRI-based neuroimaging
